# Phosphodiesterase 8B Polymorphism rs4704397 Is Associated with Infertility in Subclinical Hypothyroid Females: A Case-Control Study

**DOI:** 10.22074/ijfs.2020.6015

**Published:** 2020-07-15

**Authors:** Tabassum Mansuri, Shahnawaz D. Jadeja, Mala Singh, Rasheedunnisa Begum, Pushpa Robin

**Affiliations:** Department of Biochemistry, Faculty of Science, The Maharaja Sayajirao University of Baroda, Gujarat, India

**Keywords:** Genetic Polymorphisms, Infertility, Thyroid

## Abstract

**Background:**

Subclinical hypothyroidism (SCH) remains largely unnoticed as a major cause of infertility due to asymptomatic. Polymorphisms of phosphodiesterase 8B gene (*PDE8B*) have been linked with various diseases, including female infertility. Hence, we aimed to study prevalence of SCH, in infertile females, explore associa- tion of *PDE8B* rs4704397 A/G and rs6885099 G/A polymorphisms with infertility in females suffering from SCH and genotype-phenotype correlation of the polymorphisms with thyroid stimulating hormone (TSH) levels in Gujarat population.

**Materials and Methods:**

In this retrospective study, TSH level was estimated from plasma of 230 infertile and 100 control females by enzyme-linked fluorescence immunoassay (ELFA) to find out the prevalence of SCH. Further, based on TSH levels, thyroid function test (TFT) was performed in controls and infertile females with subclinical hypothyroidism (IF-SCH). *PDE8B* rs4704397 and rs6885099 polymorphisms were genotyped by PCR-RFLP and ARMS-PCR, respectively in 74 controls and 60 IF-SCH females.

**Results:**

We observed i. significantly high prevalence of SCH (32%) in the infertile females, ii. significant- ly lower frequency of ‘G’ allele (P=0.006), while the frequency of ‘A’ allele (P<0.0001) was higher in IF- SCH females, compared to the controls, for rs4704397 A/G SNP, iii. no significant difference in the genotype (P=0.214; OR=2.51; CI=0.74–8.42) and the allele frequency (P=0.129; OR=1.51; CI=0.92-2.47) of rs6885099 G/A SNP, iv) low linkage disequilibrium for the polymorphisms, v. significantly higher frequency of ‘AA’ hap- lotype (P=0.0001; OR=3.84; CI=1.86-8.01),while the ‘GG’ haplotype (P=0.0023; OR=0.33; CI=0.16-0.69) was significantly lower in IF-SCH females and vi. no significant difference in the TSH level of IF-SCH females with respect to the genotypes.

**Conclusion:**

The present study reports an association of *PDE8B* rs4704397 polymorphism with infertility in SCH females. The study categorically shows a higher prevalence of SCH in infertile females of Gujarat and advocates the importance of screening for SCH in infertility management.

## Introduction

Apart from its multiple functions, thyroid hormones play crucial role in reproduction. Hence, altered thyroid hormone levels can greatly affect reproductive function (1). Thyroid diseases in women with reproductive age are very common due to the complex interplay of various hormones (2). Abnormal thyroid functions of hyper or hypothyroidisms are symptomatic and they may have an adverse effect on the reproductive health contributing to infertility (3-4). However, subclinical hypothyroidism (SCH) is silent and hence it is often undiagnosed. It is a common thyroid disorder often found to coexist with various other morbidities. It is an asymptomatic condition where the patient has a normal serum free T_4_ (fT_4_/thyroxin) levels, but high thyroid stimulating hormone/thyrotropin (TSH) levels (5). TSH is considered as a sensitive indicator of the thyroid status and SCH. Normal TSH levels in serum are finely regulated in humans. Nevertheless, serum thyroid parameters show substantial inter- individual variability (6), in which genetic variations are proved as the major factors in several populations. It has been shown that altered TSH levels are related to genetic factors in up to 65% of the cases (7-9)

Different cohort studies reported phosphodiesterase 8B (*PDE8B*) as a genetic modulator of TSH levels. PDE8B gene encodes a cyclic adenosine monophosphate (cAMP) specifi c phosphodiesterase (PDE) enzyme (10). *PDE8B* affects cAMP levels in the thyroid gland resulting in changes in the levels of thyroid hormones, which in turn affects the release of TSH from the pituitary gland. *PDE8B* is mainly expressed in thyroid and brain (11, 12). Several single nucleotide polymorphisms (SNPs) for PDE8B have been demonstrated to associate with increased levels of serum TSH. More than 360,000 SNPs were tested for their associations with serum TSH levels with an additive model. The obtained results revealed three SNPs (i.e. rs4704397, rs6885099 and rs2046045) with genome-wide significance (P<10^-10^). These three SNPs were reported to be in strong linkage disequilibrium. Of the three SNPs, rs4704397 showed strongest association and it could explain 2.3% of the variations in TSH levels (13). *PDE8B* rs4704397 polymorphism has been found to associate with myocardial infarction, height (14), pregnancy (15, 16), recurrent miscarriage (17) and obesity in children (18), apart from thyroid function. Another *PDE8B* polymorphism, rs6885099 has also been shown to increase TSH levels, but to a lesser extent, in different populations (13). The relevance of human reproduction to PDE has been well-documented (19-22). While the underlying mechanism regulating oocyte maturation is not clearly known yet, the second messenger cyclic adenosine monophosphate (cAMP) role in oocyte maturation is well known (23) and thus research investigating the role of rs4704397 in the oocyte maturation might give an insight to primary infertility caused by hypothyroidism.

Numerous studies have reported the importance of screening for SCH, and the worldwide prevalence of SCH in infertile-females has been reported to be as high as 26.7% in various populations (24-27). In India, prevalence of SCH is high and reported to be 25% (28-33). However, there is no study on the status of SCH per se or its prevalence amongst infertile females in western part of India. Furthermore, there is no report on the role of PDE8B polymorphisms in female infertility. We therefore, aimed to estimate the prevalence of SCH in infertile females and explore association of *PDE8B* rs4704397 and rs6885099 polymorphisms in infertile females of Gujarat population.

## Materials and Methods

### Study subjects

The present retrospective study is a matched, casecontrol study. Two hundered and therty infertile females were recruited from Dr. Mahesh Pandya’s Ghanshyam Clinic (a fertility management center; Vadodara, India) along with 100 control females recruited from various health check-up camps. Random sampling method was followed for selection of the groups. The study protocol was explained and informed consent was obtained from all participants of the study. Seventy four out of 230 infertile females were found to have (IF) for the TSH level with the inclusion criteria of primary infertility diagnosis and duration of more than one year of unprotected intercourse without pregnancy, while 76 out of 100 controls were found to be euthyroid (with normal thyroid hormone levels). Exclusion criteria were male factor infertility, any tubal anomaly congenital or urogenital tract anomaly and history of thyroid disease/medication/surgery.

For this study, IF-SCH females/case group are defined as the infertile females who have subclinical hypothyroidism with no other clinical difficulty. In addition, they should not be under any type of medication, including thyroid disorder. Whereas, the control group includes fertile, perous, healthy euthyroid females with no medical history for thyroid or any other disorder. Control group does not include any subclinical hypothyroid female.

Sample size for the present study was calculated using G-Power software with Alpha 0.05 and effect size of 0.9. The effect size was calculated based on the observed genotype frequencies (34).

Thyroid function test Five ml blood samples was collected by venous puncture from fasting individuals and serum was separated for thyroid function test (TFT). Estimation of serum TSH, free T_3_ (fT_3_) and fT_4_ were carried out by enzyme-linked fluorescence immunoassay (ELFA) on mini VIDAS® immuno-analyzer (BioMérieux India Pvt. Ltd., India). Females having TSH values between 3.5 and 10 μIU/ml with normal fT_4_, along with an opinion from gynecologist and endocrinologist were considered as IF-SCH females. Fertile females having TSH values within the normal/euthyroid range (i.e. 0.35-3.5 μIU/ml) and fT_4_ levels within the normal range were included as controls in the present study. The reference range for serum thyroid hormones (fT_3_ and fT_4_) and TSH levels for different conditions are shown in Table S 1 (See Supplementary Online Information at www.celljournal.org). The confounding variables such as age, body mass index (BMI), smoking and hemoglobin (Hb) levels showed no significant difference between control and IF-SCH females ( Table S2, See Supplementary Online Information at www.celljournal.org).

### Genotyping *PDE8B* rs4704397 and rs6885099 polymorphisms

DNA was extracted from peripheral blood mononuclear cells (PBMCs) using ‘IAamp DNA Blood Kit (QIAGEN Inc., USA) as per manufacturer’s instructions. PDE8B rs4704397 A/G genotyping was done by polymerase chain reaction-restriction fragment length polymorphism (PCR-RFLP) while PDE8B rs6885099 (G/A) genotyping was done by amplification refractory mutation system (ARMS)-PCR. Amplification was performed using Mastercycler Gradient PCR (Eppendorf, Germany) according to the following protocol: initial denaturation at 94°C for 10 minutes, followed by 30 cycles of denaturation at 94°Cfor 45 seconds, annealing at 60°C for 45 seconds and 72°C for 1 minute. The amplified products were analyzed by electrophoresis in a 2.0% agarose gel stained with ethidium bromide. The respective primers and restriction enzyme (RE) used for genotyping are shown in Table S3. 15 μl of the amplified products was digested for 16 hours at 37°C, using 1 U restriction enzyme. For PCR-RFLP based genotyping, the digested products (300 bp and 219 bp) with 100 bp DNA ladder (Bioron, Germany) were loaded in 3.5% agarose gels stained with ethidium bromide and visualized under UV transilluminator. Furthermore, genotyping of PDE8B rs6885099 G/A was done by Amplification refractory mutation system (ARMS-PCR) in 60 IF-SCH females and 76 control females. Human growth hormone (HGH) was used, as a reaction control in the ARMS-PCR (35). Amplification was performed using Mastercycler Gradient PCR according to the following protocol: initial denaturation at 94°C for 10 minutes, followed by 35 cycles of 94°C for 30 seconds, primer dependent annealing for 30 seconds and 60°C for 1 minute. The amplified products were analyzed by electrophoresis in a 3.5% agarose gel stained with ethidium bromide using 100 bp DNA ladder.

### Statistical analysis

Hardy-Weinberg equilibrium (HWE) test was evaluated for the polymorphisms using chi-square test equating the observed and expected genotype frequencies. The genotype and allele risk associations were calculated by chisquare test using Prism 5 software (GraphPad Software Inc, USA; 2007). For genetic analysis, Bonferroni's correction was applied and statistical significance was considered at P-value less than 0.025. The linkage disequilibrium (LD) and haplotype analysis were carried out using http://analysis.bio-x.cn/myAnalysis.php (36). Levels of TSH and thyroid hormones were analyzed by non-parametric unpaired t-test and one-way ANOVA using Prism 5 software (GraphPad Software Inc.; 2007).

### In-silico analysis

Web-based in-silico prediction tool HaploReg v4.1 (https://www.pubs.broadinstitute.org/mammals/haploreg/ haploreg.php) was employed to predict the effect of noncoding rs4704397 polymorphism. Tissue specific effect of rs4704397 was assessed by an eQTL database-GTeX portal (https://www.gtexportal.org).

### Ethical consideration

It was ensured that the study design complies with the ethical standards of the Institutional Ethical Committee for Human Research (IECHR), Faculty of Science, The Maharaja Sayajirao University of Baroda,Vadodara, Gujarat, India (FS/IECHR/BC/PR/1) and with the 1964 Hel inki declaration.

## Results

### Estimation of thyroid stimulating hormone, free T3 and free T4 levels

**Fig 1:**
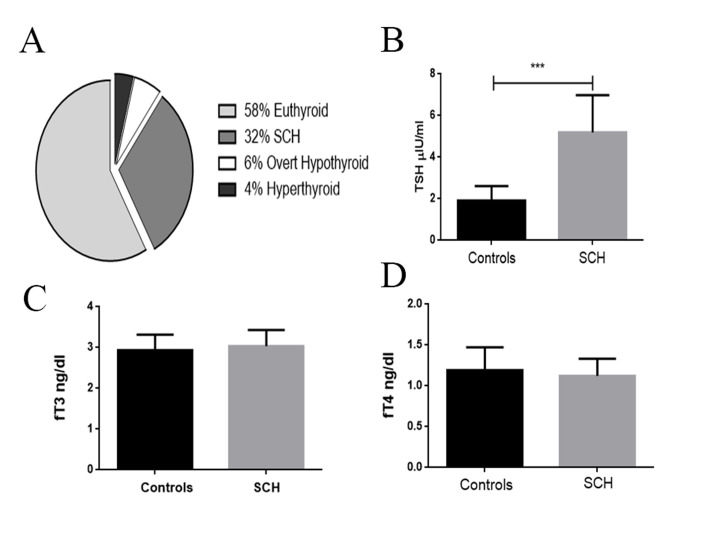
Estimation of TSH and thyroid hormone levels. **A.** Prevalence of thyroid dysfunction among the infertile females. **B.** TSH level in controls and IF-SCH females. **C.** fT_3_ levels in the controls and IF-SCH females. **D.** fT_4_ levels in controls and IF-SCH females. TSH; Thyroid stimulating hormone, IF-SCH; Infertile females with subclinical hypothyroidism, fT_3_; Free T_3_, fT_4_; and Free T_4_.

Analysis of TSH, fT_3_ and fT_4_ levels in the studied subjects revealed that among 230 females with primary infertility, 58% (n=133) were euthyroid, 32% (n=74) were SCH, 6% (n=14) were overt hypothyroid and the rest 4% (n=9) females were hyperthyroidism (Fig .1 A, Table S3) (See Supplementary Online Information at www. celljournal.org). IF-SCH females had significantly higher (P<0.0001; Fig.1B) TSH levels (mean ± SEM: 5.34 ± 0.21 μIU/ml) compared to the control females (mean ± SEM: 1.91 ± 0.08 μIU/ml) and they had no significant difference in fT3 levels (P=0.1159, mean ± SEM: 3.036 ± 0.0462pg/ml; Fig.1C) compared to the controls (mean ± SEM: 2.935 ± 0.0436). There was no significant difference between fT4 levels (P=0.0741, mean ± SEM: 1.22 ± 0.0249) in IF-SCH females compared to controls (mean ± SEM: 1.195±0.0318 ng/dl).

### *PDE8B* rs4704397 SNP in infertile females with subclinical hypothyroidism females

**Fig 2:**
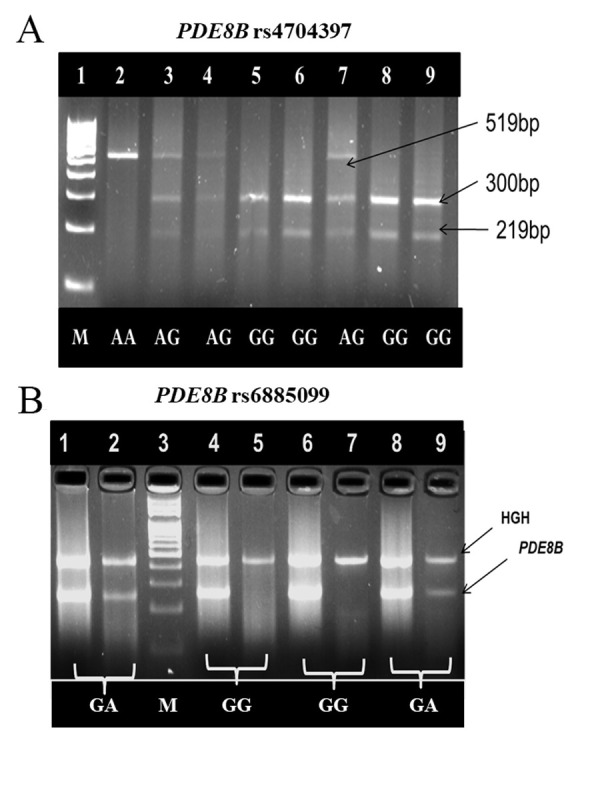
Representative gel images for PDE8B rs4704397 and rs6885099 genotyping. **A.** PCR-RFLP analysis of PDE8B rs4704397 SNP on 3.5% agarose gel. Lane 1 shows 100 bp ladder, lane 2 shows homozygous (AA) genotype, lanes 3, 4 and 7 show heterozygous (AG) genotypes, lanes 5, 6, 8 and 9 show heterozygous (GG) genotypes. **B.** ARMS-PCR analysis of PDE8B rs6885099 SNP on 3.5% agarose gel. Lanes 1 and 2 show homozygous (GA); lane 4, 5, 6 and 7 show homozygous (GG) genotypes and lane 3 shows 100 bp ladder, lanes 8 and 9 show heterozygous (GA) genotypes. PCR-RFLP; Polymerase chain reaction-restriction fragment length polymorphism.

Genotyping *PDE8B* rs4704397 polymorphism was carried out in 60 IF-SCH females and 76 healthy fertile females (Fig .2A). Other variables such as age (P=0.419), BMI (P=0.309), smokers (0%) and Hb (P=0.117) levels were not significantly different between the subjects of each genotypes ( Table S4). The observed genotype frequencies of *PDE8B* rs4704397 SNP in IF-SCH females were slightly deviated from HWE (P=0.049; Table 1), whereas the control population was under HWE (P=0.062; Table 1). Ancestral allele ‘A' and genotype ‘AA’ were considered as the reference allele and genotype respectively. The frequency of AG and GG genotypes were significantly lower in IF-SCH females, compared to controls (P=<0.0001 and P=0.006 respectively; Table 1). The frequency of ‘G’ allele was also significantly lower in IFSCH females, compared to the control females (23% vs. 47%, P<0.0001, OR=0.34). Hence, “G” allele was identified to have a protective effect and ‘A’ allele was identified as the risk allele for SCH and infertility in females.

### *PDE8B* rs6885099 SNP in infertile females with subclinical hypothyroidism

Genotyping of *PDE8B* rs6885099 polymorphism was carried out in 60 IF-SCH and 76 control females (Fig .2B). The observed genotype frequencies of *PDE8B* rs6885099 polymorphism among the control and IF-SCH females were in accordance with HWE (P=0.248 and P=0.134 respectively; Table 2). Distribution of genotype as well as allele frequencies revealed no significant difference among the IF-SCH and control females (Table 2).

**Table 1:** Distribution of genotype and allele frequencies for PDE8B rs4704397 A/G polymorphism


Genotype or allele	IF-SCH females(Freq. %)	Control females(Freq. %)	P value	Odds Ratio	95% CI	P value HWE

Genotype	n= 60	n=76				
AA	38 (63%)	17 (22%)	R	1	0.07-0.35	0.062 (C)
AG	16 (27%)	46 (61%)	<0.0001^a^	0.16	0.07-0.63	
GG	06 (10%)	13 (17%)	0.006^a^	0.21		0.049 (P)
Allele						
A	92 (77%)	80 (53%)	R	1	-	
G	28 (23%)	72 (47%)	<0.0001^b^	0.34	0.19-0.57	


n; number of IF-SCH females/control females, R; reference group, Freq.; Frequency, CI; Confidence interval, P; IF-SCH females, C; Control females, a IF-SCH female vs. control females (genotype) using chi-squared test with 2×2 contingency table, and b IF-SCH females vs. control females (allele) using chi-squared test with 2×2 contingency table, and IF-SCH; Infertile females with subclinical hypothyroidism.

**Table 2:** Distribution of genotypes and alleles for PDE8B rs6885099 G/A polymorphism


Genotype or allele	IF-SCH females(Freq. %)	Control females(Freq. %)	P value	Odds Ratio	95% CI	P value HWE

Genotype	n= 60	n=76				
GG	17 (28%)	32 (42%)	R	1	-	-
GA	35(58%)	38 (50%)	0.1914^a^	1.73	0.82-3.65	0.248 (C)
AA	08 (13%)	06 (8%)	0.2145^a^	2.51	0.74-8.42	
Allele						0.134 (P)
A	69 (58%)	102 (67%)	R	1	-	
G	51 (42%)	50 (33%)	0.1292^b^	1.51	0.92-2.47	


F-SCH; Infertile females with subclinical hypothyroidism; n; number of IF-SCH females/Control females, R; reference group, Freq.; Frequency, CI; Confidence interval, P; IF-SCH females and C; Control females, ^a^ IF-SCH female vs. control females (genotype) using chi-squared test with 2×2 contingency table, and ^b^ IF-SCH females vs. control females (allele) using chisquared test with 2×2 contingency table.

**Table 3:** Distribution of haplotype frequencies for PDE8B rs4704397 and rs6885099 polymorphisms


Haplotype[rs4704397(A/G):rs6885099 (G/A)]	IF-SCH FemaleFreq. (%)	Control femalesFreq. (%)	P value for association	P value (Global)	Odds Ratio [95% CI]

AG	48 (46%)	49 (21%)	0.4434	7.5 × 10^-5^	1.230 [0.72-2.09]
AA	31 (30%)	12 (10%)	0.0001		3.84 [1.86-8.01]
GG	12 (12%)	34 (28%)	0.0023		0.33 [0.160-0.69]
GA	13 (12%)	25 (21)	0.0876		0.53 [0.25-11.10]


Freq.; Frequency, CI; Confidence interval (Frequency <0.03 in both control and case has been dropped and it was ignored in the analysis), and IF-SCH; Infertile females with subclinical hypothyroidism

#### Linkage disequilibrium and haplotype analysis

Linkage disequilibrium (LD) analysis revealed that two investigated PDE8B polymorphisms (i.e. rs4704397 and rs6885099) were in low LD association (D’=0.060, r2=0.003). Haplotype analysis revealed that the frequency of ‘AA’ haplotype was significantly higher in the patients and risk of IF-SCH females was increased by 3.84 fold (P=0.0001, OR=3.84; CI=1.86-8.01; Table 3). The frequency of ‘GG’ haplotype was significantly lower in IF-SCH females, compared to the controls suggesting its protective effect (P=0.0023, OR=0.33; CI=0.16-0.69; Table 3).

### Genotype-phenotype correlation analysis

**Fig 3:**
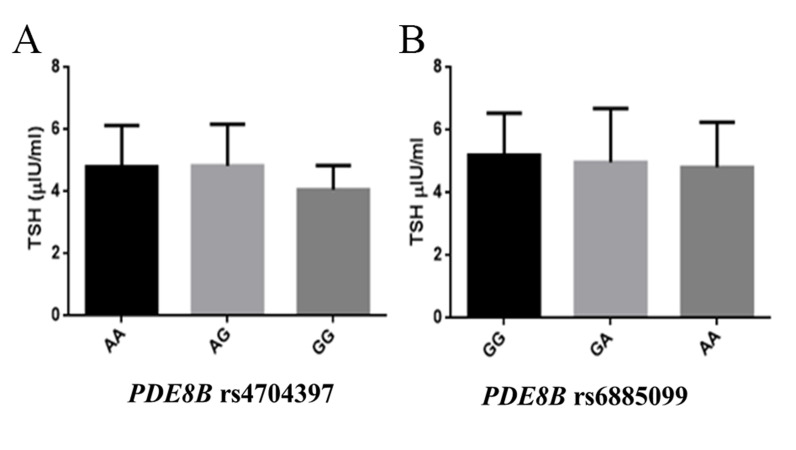
Correlation of *PDE8B* rs4704397 and rs6885099 with TSH levels in IF-SCH females. No significant difference of TSH levels was observed with respect to PDE8B polymorphisms **A.** rs4704397 and **B.** rs6885099. TSH; Thyroid stimulating hormone, IF-SCH; Infertile females with subclinical hypothyroidism

TSH levels in IF-SCH females were analyzed with respect to the genotypes of *PDE8B* rs4704397 A/G and rs6885099 G/A. No significant difference in TSH levels was observed with respect to genotypes of the both SNPs (Fig .3).

### In-silico analysis

Analysis of functional consequences of PDE8B rs4704397 by HaploReg v4.1 predicted that PDE8B rs4704397 could alter heat shock factor-type (HSF) motif and enhancer state by H3K27 acetylation (H3K27ac) in inferior temporal lobe of brain (https:// www.pubs.broadinstitute.org/mammals/haploreg/detail_ v4.1.php?query=&id=rs4704397).eQTL database GTEx portal showed significantly elevated PDE8B transcripts in thyroid tissue of individuals carrying ‘A’ allele, compared to ‘G’ allele (https://www.gtexportal.org/home/snp/ rs4704397).

## Discussion

The present study shows a high prevalence rate of SCH in infertile females (32%) in comparison with the healthy controls (Table S1) and the association of rs4704397 SNP with infertility in IF-SCH females of Gujarat region. In developing countries, one among four couples suffers from infertility and in these couples, hypothyroidism is one of the key perpetrators. In a study performed by Verma et al. (28), out of 394 infertile women, 23.9% were hypothyroid (TSH>4.2 μIU/ml). An intervention to rectify the hypothyroidism resulted in 76.6% of the conceived infertile women. Primary health caregivers most often pick up overt hypothyroidism easily; however, SCH with its subtle symptoms most often goes unnoticed. The prevalence of SCH amongst infertile females is common, but there is a scarcity on available data. However, there are a few studies reporting the prevalence of hypothyroidism, ranging from 15-25% in Indian population (28- 33). As SCH is largely asymptomatic, it goes undiagnosed, resulting in infertility. It is essential to include evaluation of thyroid related hormones as a standard practice along with other tests to ascertain the causes of infertility.

SCH occurs due to multiple factors. Some of them include congenital agenesis, defect in synthesis due to iodine deficiency or anti-thyroid drugs, autoimmune diseases, post-surgery, hypopituitarism, TSH deficiency, environmental pollutants, mutations and SNPs (37). Of these factors, the present study focuses on the SNPs. To evaluate possible correlation between the polymorphisms associated with increased TSH levels and infertility, two SNPs (rs4704397 and rs6885099) of the *PDE8B* were studied in healthy controls and IF-SCH females. Higher frequency of the “A” allele for *PDE8B* rs4704397 polymorphism in SCH related infertile patients which revealed “A” as a risk allele for infertility in IF-SCH females. However, *PDE8B* rs6885099 was not associated with infertility. Earlier, *PDE8B* rs4704397 was also found to associate with recurrent miscarriage (17). *PDE8B* is found in the thyroid but not pituitary. In addition, given the importance of cAMP activity in TSH signaling, it is suggested that the *PDE8B* rs4704397 polymorphism could reduce cAMP levels in the thyroid resulting in a decreased response of thyroid gland to TSH stimulation, which leads to an increase of TSH set point for the same free T3 and T4 levels (18). Polymorphism in *PDE8B*, rs4704397 results in an increase in *PDE8B* enzyme expression. We propose that this could result in a faster degradation of cAMP, which decreases the synthesis and release of T3 and T4. In such a scenario, the negative inhibition of Thyrotropin-releasing hormone (TRH) will not take place and this will result in increased levels of TRH and hence TSH. As a consequence, T3 and T4 levels become normal. The increased level of TSH results in development of SCH. *PDE8B* rs4704397 polymorphism might induce phosphodiesterase activity in *PDE8B*, thereby reducing the ability of thyroid gland to generate free T4 when stimulated by TSH. This results in SCH, which can be the cause of infertility in IF-SCH patients. Arnaud et al. in a GWAS study reported that *PDE8B* rs4704397 could affect plasma TSH levels. Each copy of the minor allele “A” may lead to a mean increase of 0.13 mU/l TSH levels (13). However, we did not observe significant correlation of the *PDE8B* rs4704397 SNP with circulating TSH levels. This might be due to the limited sample size in the present study. *PDE8B* rs4704397 SNP was also found to be associated with various conditions like cardiovascular, body height, pregnancy, recurrent miscarriage, obesity in children, etc. (14-18). Though the exact underlying mechanism of *PDE8B* rs4704397 SNP affecting TSH levels is not clear, in-silico tools predicted that this variation might lead to enhancement of *PDE8B* expression by influencing epigenetic level. The role of PDE8B in human placenta and ovaries is still to be understood, while human reproduction relevance to PDE has been proposed (19-22). The underlying mechanism of regulating oocyte maturation is not clearly documented yet, but the second messenger cAMP role in oocyte maturation is well known (23). Thus, investigating the role of rs4704397 in the oocyte maturation could be an interesting area of research as far as female infertility is concerned.

On the other hand, medications given to alter the levels of reproductive hormones have serious repercussions on the health of females with long-term implications (38). Treatment of infertility is usually done by direct targeting the reproductive system, instead of looking for the involvement of other factors, such as genetic polymorphisms, as a cause of infertility. This genetic approach could be used to identify IF-SCH patients and treat infertility with greater success and fewer side-effects without disturbing the reproductive system. Since, small sample size was a limiting factor for the present study, we suggest investigating larger number of infertile females in different populations. This might provide a significant insight into understanding the role of *PDE8B* in infertility.

## Conclusion

The present study establishes an association of PDE8B rs4704397 with infertility in IF-SCH females and reiterates the importance of screening SCH, as a diagnostic tool in infertility management.

## Supplementary PDF


