# Supplementary material for: Phosphodiesterase 8B Polymorphism rs4704397 Is Associated with Infertility in Subclinical Hypothyroid Females: A Case-Control Study
Source: Int J Fertil Steril. 2020 Jul 15;14(2):122–9. doi: 10.22074/ijfs.2020.6015 (PMC7382679; doi:10.22074/ijfs.2020.6015)
Supplement: Supplementary file 1 [file Int-J-Fertil-Steril-14-122-s01.pdf]

Int J Fertil Steril
International Journal of Fertility & Sterility
ISSN:2008-076X eISSN:2008-0778

Supplementary Information for

Phosphodiesterase 8B Polymorphism rs4704397 is Associated with Infertility in Subclinical Hypothyroid Females: A Case-Control Study

Tabassum Mansuri, M.Sc., Shahnawaz D. Jadeja, M.Sc., Mala Singh, Ph.D., Rasheedunnisa Begum, Ph.D., Pushpa Robin, Ph.D.*

Department of Biochemistry, Faculty of Science, The Maharaja Sayajirao University of Baroda, Vadodara-390002, Gujarat, India.

Table S1: Reference range of TSH, fT_3 and fT_4 levels followed for the selecting the subjects.

Likely Diagnosis	TSH Levels (µIU/ml)	fT_3(pg/ml)	fT_4(ng/dl)
Euthyroid	0.35-3.5	2.3-4.2	0.9-1.76
Subclinical hypothyroidism	3.5-10 (High)	Normal	Normal
(SCH)	> 10	Low	Low
Hypothyroid	< 0.35	High	High
Hyperthyroid	< 0.35	High	High

TSH; thyroid stimulating hormone, fT_3; Free T_3, and fT_4; Free T_4; number of IF-SCH females

Table S2: Demographic details

Study subjects	Control females	Infertile females
Total, no.	100	230
Age, years (Mean ± SEM)	30.75 ± 0.63	28.16 ± 0.57
BMI, kg/m^2(Mean ± SEM)	23.26 ± 0.27	23.62 ± 0.25
Hb, gm/dl (Mean ± SEM)	11.65 ± 0.19	12.07 ± 0.14
Smokers (%)	0	0
Euthyroid, no.(%)	76 (76)	133 (58)
SCH, no. (%)	8 (8)	74 (32)
Overt Hypothyroid, no. (%)	10 (10)	14 (6)
Hyperthyroid, no. (%)	6 (6)	9 (4)

BMI; Body mass index, SCH; Subclinical hypothyroidism, and Hb; Smoking and hemoglobin

Table S3: Primers and restriction enzyme (RE) used for genotyping PDE8B rs4704397 and rs6885099 polymorphisms

Gene	SNP	Primers	Product	RE	Cut products
PDE8B	rs4704397	FP: GGCGCTACTCTAGGTTTGGA<br>RP: GTCTGCTCCTTGGCTTTTCC	519bp	Bsl I	300bp, 219bp
PDE8B	rs6885099	FP1:GTTCAAGAGGACTTTACCGAG<br>FP2:GTTCAAGAGGACTTTACCGAA<br>RP:ACCTGGTCTGGACGTCAG	231bp	-	
HGH	-	FP: TCAGTTCCTCCTTACTCATGG<br>RP: CACCTGTAACTGGCTGTTTG	428bp	-	

Table S4: Clinical characteristics of the studied population according to the *PDE8B* rs4704397 genotypes

	AA	AG	GG	P-value (ns)
Age, years	29.39 ± 0.73	30.43 ± 0.45	30.20 ± 0.74	0.419
BMI, Kg/m^2	23.92 ± 0.30	23.41 ± 0.29	23.18 ± 0.42	0.309
Hb g/dl	11.47 ± 0.30	12.09 ± 0.16	11.53 ± 0.30	0.117

Data represents Mean ± SEM values, BMI; Body mass index, and Hb; Hemoglobin.

Received: 14/June/2019, Accepted: 22/December/2019
*Corresponding Address: Department of Biochemistry, Faculty of Science, The Maharaja Sayajirao University of Baroda, Vadodara-390002, Gujarat, India
Email: pushparobin@gmail.com
